# Socioeconomic status and subjective social status measurement in KiGGS Wave 2

**DOI:** 10.17886/RKI-GBE-2018-033

**Published:** 2018-03-15

**Authors:** Thomas Lampert, Jens Hoebel, Benjamin Kuntz, Stephan Müters, Lars Eric Kroll

**Affiliations:** Robert Koch Institute, Berlin, Department of Epidemiology and Health Monitoring

**Keywords:** SOCIOECONOMIC STATUS, SUBJECTIVE SOCIAL STATUS, METHODS, HEALTH MONITORING, KIGGS

## Abstract

This article describes the method applied to measure socioeconomic status (SES) and subjective social status (SSS) in the current wave of the German Health Interview and Examination Survey for Children and Adolescents (KiGGS Wave 2), which was conducted over three years between 2014 and 2017. The composite multidimensional SES index was calculated as a sum of point scores for the parents’ education level, occupational status and equivalised disposable income. SSS was assessed in the 11 to 17 year age group using a German version of the MacArthur Scale for children and adolescents. To demonstrate the use of both instruments, we present examples that highlight the association between SES and SSS with the general health of children and adolescents in the 3 to 17 and/or 11 to 17 age groups. Over 95% of parents rated the general health of their children as ‘very good’ or ‘good’. However, the analyses clearly reveal that children and adolescents from families with low SES and SSS have poorer general health than their better-off peers. Even when mutually adjusted, both low SES and SSS are independently associated with poorer general health. In addition to the SES index, studies on the health of children and adolescents should therefore also consider SSS. In this way, additional aspects of the socioeconomic conditions of families can be taken into account.

## 1. Introduction

Numerous national and international studies have shown the close link between child and adolescent health and the socioeconomic status of the families they grow up in [1-6]. Data from the baseline study of the German Health Interview and Examination Survey for Children and Adolescents (2003-2006) and the subsequent KiGGS Wave 1 (2009-2012) [7-9] conducted by the Robert Koch Institute (RKI) have also highlighted this fact. As these studies indicate, children and adolescents from low socioeconomic status backgrounds have significantly poorer health compared to their peers from socioeconomically more affluent families. This fact reveals itself in the general state of health and in psychosocial health, for example with regard to behavioural problems, attention deficit hyperactivity disorder (ADHD) and eating disorders [6, 7, 9-11]. Furthermore, social differences in health behaviours are evident, for example regarding tobacco consumption, physical activity and dietary habits. The same applies to healthcare system utilisation, as much regarding the use of medical services, as also preventive healthcare and health promotion services [6, 9, 12-14].

In the KiGGS study, a composite index is used to measure socioeconomic status, which is based on information regarding the parents’ education, occupational status and income [15]. This ‘index of socioeconomic status’ (SES index) is used in a comparable manner in all of the established health monitoring surveys at the RKI [16]. The only difference is that in the KiGGS study, the status determining data is collected from the participants’ parents, whereas in the RKI’s surveys among adults, participants self-report this data. The comparable and consistent construction of the SES index in the surveys conducted at the RKI make it possible to relate the results of the surveys and to analyse trends over time. Meanwhile, many other epidemiologic studies in Germany have been applying the SES index, as much to study child and adolescent health as well as the health of middle-aged and elder adults [17].

**KiGGS Wave 2**

Second follow-up to the German Health Interview and Examination Survey for Children and Adolescents

**Data owner:** Robert Koch Institute

**Aim:** Providing reliable information on health status, health-related behaviour, living conditions, protective and risk factors, and health care among children, adolescents and young adults living in Germany, with the possibility of trend and longitudinal analyses

**Study design**: Combined cross-sectional and cohort study

**Cross-sectional study in KiGGS Wave 2**

**Age range:** 0-17 years

**Population:** Children and adolescents with permanent residence in Germany

**Sampling:** Samples from official residency registries - randomly selected children and adolescents from the 167 cities and municipalities covered by the KiGGS baseline study

**Sample size:** 15,023 participants

**KiGGS cohort study in KiGGS Wave 2**

**Age range:** 10-31 years

**Sampling:** Re-invitation of everyone who took part in the KiGGS baseline study and who was willing to participate in a follow-up

**Sample size:** 10,853 participants

**KiGGS survey waves**

► KiGGS baseline study (2003-2006), examination and interview survey
► KiGGS Wave 1 (2009-2012), interview survey
► KiGGS Wave 2 (2014-2017), examination and interview survey

More information is available at www.kiggs-studie.de/english

In addition to the SES index, the health surveys conducted at the RKI will in future also assess subjective social status (SSS), which measures a participant’s subjective perception and assessment of their socioeconomic situation [18-20]. SSS can have independent health implications above and beyond the effects of objective SES, which can be observed not only in adulthood, but also in adolescence [21-23].

In the following sections, we provide a detailed description of how the socioeconomic variables were operationalised in the KiGGS Wave 2 study and how the SES index was designed and generated. Moreover, we describe how SSS was measured in this survey. Subsequently, we provide examples of results that reveal the association of both, SES index and SSS with general health among children and adolescents aged 3 to 17 and 11 to 17 years. The results aim to show the extent of socioeconomic differences in general health among children and adolescents in Germany. Moreover, they provide insights regarding whether the SES index and SSS are each independently associated with child and adolescent health.

## 2. Methods

### 2.1 Data basis and statistical analysis

As part of health monitoring at the RKI, KiGGS is a central source of information to assess the health of the adolescent generation in Germany [24, 25]. For the 0 to 17 age group, KiGGS regularly provides representative cross-sectional data. Furthermore, the KiGGS cohort, which has been designed as a longitudinal follow-up study, interviews and examines the participants in the KiGGS baseline study repeatedly up to adult age.

The KiGGS baseline study (2003-2006) consisted of interviews, examinations and laboratory analyses. In KiGGS Wave 1 (2009-2012), data was collected by telephone interviews [26]. 17,641 children and adolescents aged 0 to 17 from 167 locations in Germany took part in the KiGGS baseline study. The response rate was 66.6% [27]. The KiGGS Wave 2 (2014-2017) sample consisted of a new sample from the population registry of the original 167 KiGGS baseline study sample points (Infobox). KiGGS Wave 2 therefore comprises a new nationwide cross-sectional survey for 0 to 17 year-old children and adolescents in Germany and the second follow-up of the KiGGS cohort [28]. A total of 15,023 children and adolescents (7,538 girls, 7,485 boys) aged 0 to 17 took part in the KiGGS Wave 2 cross-sectional survey (response rate 40.1%) [29].

All surveys at the Robert Koch Institute are subject to strict compliance with the data protection regulations of Germany’s Federal Data Protection Act. The Hannover Medical School ethics committee has considered and approved the survey under ethical guidelines (No. 2275-2014). The Federal Commissioner for Data Protection and Freedom of Information in Germany had no objections to the study. Participation in the study was voluntary. Participants, their parents and/or legal guardians were informed about the objectives and content of the study and data protection, and provided their informed consent in writing.

The analyses of the relationship between socioeconomic status and/or subjective social status and the general health of children and adolescents are based on parents’ assessment of the health of their children [30]. As recommended by the World Health Organization, parents were asked [31]: ‘How would you rate your child’s health in general?’ (Answer categories: very good, good, fair, bad and very bad). The results concern children and adolescents aged 3 to 17 (n=13,568). For subjective social status, the results concern participants aged 11 to 17 (n=6,599), because SSS was not assessed in younger children. The results reflect prevalence rates, as well as, through binary logistic regression, age-adjusted odds ratios. The odds ratios presented express the degree to which a specific group has a higher statistical odds of fair, bad or very bad health compared to the defined reference group.

Weighting factors are used to account for unequal sampling probabilities and to adjust the distribution of the sample by age, gender, region, nationality and level of parental education to match the official German population statistics for 2014/2015 and the 2013 microcensus. Additionally, the weighting factor adjusts for differences in the rate of participants in the KiGGS baseline study and KiGGS Wave 1, who took part again [28]. To account for weighting and correlation of participants within one municipality, confidence intervals and logistic regression models are calculated using procedures for complex samples. Differences are considered statistically significant when p-values are lower than 0.05. All analyses are conducted with the statistics software Stata 14.2 SE.

### 2.2 Operationalisation of the SES index

In KiGGS Wave 2, the three dimensions education, occupation and income, which are generated as household characteristics based on the data provided by parents, are used to determine SES. Operationalisation of the index is comparable to the method first used in KiGGS Wave 1 (on the operationalisation of the three status dimensions see Annex Table 1). In the KiGGS baseline study, the index was initially developed differently, but was later re-calculated based on the new template, so that the results from KiGGS Wave 1 and KiGGS Wave 2 are now comparable to baseline study results [15].

For indexing, point scores are calculated for each status dimension (see Annex Table 2). Regarding education and occupation, the SES index registers the highest point score a child’s parents provide. Only children who lived in exclusively single-parent households without their partner are assigned the score of their single parent directly. Scores for each dimension ranged from 1 to a maximum of 7. The sum of point scores from the individual dimensions become equal parts of the SES index.

As the first SES dimension, levels of education are assigned based on the international CASMIN (Comparative Analysis of Social Mobility in Industrial Nations) classification [32]. This classification distinguishes nine levels of education, which are defined as distinct combinations of school degrees and vocational qualifications. The standardised point scores used in the SES index range between 1 and 7 reflect the average salaries people earn based on their educational attainments in Germany. A regression model is used to determine the point scores based on the gross hourly wages of the German workforce aged 30 to 59 using data from the 2013 German Socioeconomic Panel (SOEP) study. Children and adolescents are assigned the maximum point score their parents provided, except in cases, where the child lived exclusively with only one parent (without a partner).

We use Ganzeboom and Treimann’s International Socio-Economic-Index of Occupational Status (ISEI) as a criterion to assign point scores for occupational status as a second dimension of SES [33]. The ISEI index is based on occupations coded according to the 2008 International Standard Classification of Occupations (ISCO-08) [34]. The point scores included in the SES index vary between 1 and 7 and are generated based on the data provided by parents on their occupational activity. Parent occupations are coded applying a standardised procedure according to the classification of occupations of the Federal Statistics Office (2010) and then semi-automatically transferred to the ISCO-08 classification. Each child and adolescent is assigned the maximum score provided by parents, except if he or she lived exclusively with only one parent (without a partner).

Income as the third dimension of SES is measured by needs-adjusted net household income (equivalised disposable income) as an indicator in accordance with the guidelines of Germany’s federal reporting on poverty and wealth and the recommendations for reporting on social cohesion in Europe [17, 35]. In cases where parents did not provide exact salary amounts but a salary range, these salaries are evenly distributed across the corresponding interval analogous to the German Microcensus [36]. For income categories such as a range from €2,000–2,500 we so not assume the mean value of €2,125, but take distribution-based random values within this interval. Missing values for net household income are imputed through regression imputation [16]. To estimate missing values for income, data on the age of parents, their levels of education and occupational status, as well as regional information of the German Federal Statistical Office on mean net household income in the participants' residential area is used. Point scores are determined by defining 13 equally large income groups (equivalised disposable income), which are then consolidated into seven SES point scores for income. The intervals between the point scores for educational attainment and occupational status reflect equidistant intervals with regard to external criteria. We can therefore assume a metric scale for the individual SES dimensions.

### 2.3 Calculation of the multidimensional SES index and delimitation of socioeconomic groups

The SES index is calculated as a sum of point scores based on the values assigned to the three dimensions of education, occupational status and income. It is used as a household characteristic, which means that all participants in one particular household are assigned the same index value. The three equally weighted subscales of education, occupational status and income provide the basis for calculating the SES index, which means that SES index values ranged between 3.0 and 21.0. The SES index can enter the analysis as a metric variable or be categorised in various socioeconomic groups. These groups then reflect a ranking of children and adolescents with regard to their household socioeconomic status. We propose a distribution-based classification into five equally large groups (quintiles), whereby the three groups in the middle are combined. Through the accumulation of educational qualifications, occupational status and income, this three-step scale – low SES (first quintile), medium SES (second to fourth quintile) and high SES (fifth quintile) – facilitates comparisons between the 20% of children and adolescents who grow up in the most and least socioeconomically affluent families, with a broadly defined medium segment comprising the other 60% of children and adolescents. Table 1 shows the categories, cut-off points and corresponding share of participants in KiGGS Wave 2. The share of missing values was less than 1.5%.

Table 2 shows the statistical association between the SES index and its three dimensions based on correlation coefficients. The values for the overall SES index correlated with the individual dimensions between r=0.83 and r=0.85. The correlations are comparable to KiGGS Wave 1 [15], as well as to the correlations among adult participants in the German Health Update (GEDA) study [16].

### 2.4 Operationalisation of subjective social status

In health research and epidemiology, the definition of SES based on ‘objective’ status indicators such as education, occupation and income is more and more often supplemented by subjective status indicators. Whereas objective status indicators assign people to the ‘upper and lower rungs’ of society, subjective status indicators capture how people themselves view their social standing and the status group that they feel they belong to [18-20]. Objectively assigned and subjectively perceived status do not always have to coincide.

The additional collection of data on subjective social status (SSS) in epidemiologic studies adds a subjective dimension to the measurement of socioeconomic status and grants the individual perception of living conditions and relative social standing a role in health opportunities and disease risks. In recent years, evidence has been accumulated suggesting an independent effect of SSS on health and disease above and beyond the effects of objective SES [18, 19, 37-39]. The effect is thereby visible not only at adult age, but already at adolescent age [21-23, 40]. For example, it is assumed that perceptions of relative disadvantage can evoke feelings of shame, injustice or envy that cause stress and can therefore increase the risk of physical and mental health problems [41-44]. To a certain degree, a person’s subjectively perceived social status is likely to reflect aspects of their socioeconomic situation such as wealth, over-indebtedness or social security, which the traditional indicators of education, occupation and income do not capture.

Adolescence is a phase in life in which young people increasingly make their own experiences with social inequality [21, 45]. The radius of interaction with society and the contact with diverse social groups outside the family increase. Adolescents therefore increasingly compare their social situation to that of others and their perception of social disparities and the advantages and disadvantages in accessing wealth, consumption, education opportunities, power and social recognition grows. Adolescents then increasingly develop their own perception and understanding of their social status and that of their families. Subjective indicators of social status can capture these perceptions, which objective status indicators cannot account for.

In KiGGS Wave 2, SSS was measured using a German version of the MacArthur Scale for children and adolescents. Initially, the MacArthur Scale was developed to record SSS for adults in the US [18]. Recently, the Robert Koch Institute developed a German version of this scale for adults [20, 46]. The instrument uses the image of a ladder with ten rungs that represent society as a visual analogue scale. Respondents mark their subjectively felt position on this ‘social ladder’. Goodman et al. [21] have developed a version for adolescents, as the original instrument asks adults where they see themselves compared to other people from their country regarding levels of education, occupation and income. Adolescents, however, mostly still go to school, have not yet embarked on their career and are not financially independent so their status is defined by the status of their families. The version for adolescents therefore asks where adolescents would position their family on the ladder. For KiGGS Wave 2 the English language scale was translated into German and adapted for use with a German sample. The scale became part of the questionnaires for the 11 to 17 age group. The German question wording can be found in Annex Table 3.

Table 3 shows the mean MacArthur Scale values of responses in KiGGS Wave 2. The self-assessments of 11 to 17 year-olds show that the girls and boys in this age group on average position themselves slightly above the middle of the ten-rung scale. Girls mark a mean value of 6.2 and boys 6.3. The mean value for girls in the 11 to 13 age group are higher than in the 14 to 17 age group (p=0.041), a difference not observable for boys (p=0.672). More pronounced differences are observable in an analysis stratified by objective SES. Mean SSS gradually increases with higher objective family SES, both for girls (p<0.001) and boys (p<0.001). Correlation analysis reveals a clearly positive association between SSS and the objective status indicators of the SES index (Table 4). The correlation with the objective family SES index score is r=0.39 for girls and r=0.42 for boys. Among the three single SES subscores, family income shows the strongest correlations with SSS of girls and boys.

## 3. Links between socioeconomic status, subjective social status and general health

**Figure 1:**
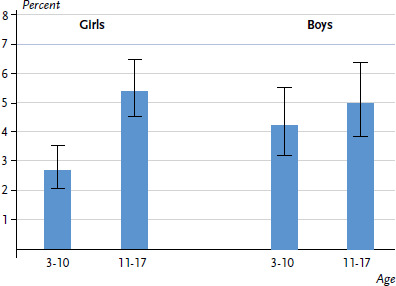
General health (fair to very bad) among girls and boys according to age group (n=6,682 girls, n=6,633 boys) Source: KiGGS Wave 2 (2014-2017)

According to the parent ratings, 2.7% of 3 to 10 year-old and 5.4% of 11 to 17 year-old girls have fair to very bad general health. The figures for boys are 4.2% of 3 to 10 year-olds and 5.0% of 11 to 17 year-olds (Figure 1) [30]. When controlled for age, no statistically significant differences are discernible between girls and boys.

**Figure 2:**
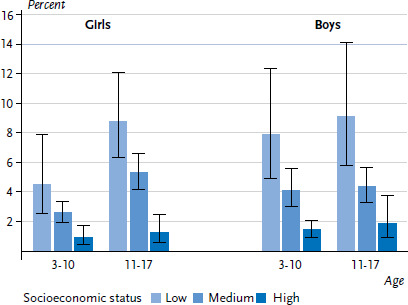
General health (fair to very bad) among girls and boys according to socioeconomic status and age group (n=6,650 girls, n=6,610 boys) Source: KiGGS Wave 2 (2014-2017)

When comparing children and adolescents against the backdrop of the socioeconomic status of their families, clear differences in general health become evident. Children and adolescents from low SES family backgrounds are more likely to have only fair to very bad general health. The share of girls in the 3 to 10 age group with fair to very bad general health is 4.5% for children in the lowest socioeconomic group, whereas it is 2.6% for those from medium and 0.9% for those from high socioeconomic backgrounds. For girls in the 11 to 17 age group, health differences related to SES are 8.8% for the low, 5.3% for the medium and 1.2% for the high socioeconomic group. Similar SES-related differences in general health are observed for boys in both age groups (Figure 2).

When the age of children and adolescents is statistically controlled for in logistic regression models, the results indicate an approximately six times increased odds of only fair to very bad general health for children and adolescents from low SES families compared to those from high SES families. For children and adolescents from the medium socioeconomic group, the odds is about three times as high compared to those from a high socioeconomic group (Table 5). However, in the high socioeconomic group, the prevalence of fair to very bad general health is very low (Figure 1 and Figure 2), which means that the high odds ratios must be interpreted with caution.

**Figure 3:**
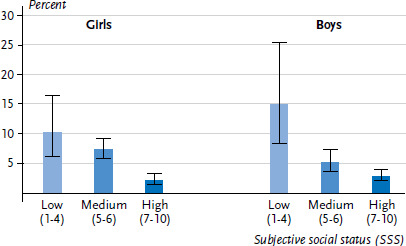
General health (fair to very bad) in the 11 to 17 age group according to subjective social status (n=3,090 girls, n=2,817 boys) Source: KiGGS Wave 2 (2014-2017)

Subjective social status (SSS) too shows a close association with the general health of children and adolescents. The prevalence of only fair to very bad general health for 11 to 17 year-old girls and boys with low SSS (scale values of 1–4) is also clearly higher than the prevalence for those with high SSS (scale values of 7–10, Figure 3). Controlled for age, the odds of fair to very bad general health in the low SSS group is five and a half times higher than in the high SSS group. In the medium SSS group, the odds is about two and half times higher (Table 6).

**Figure 4:**
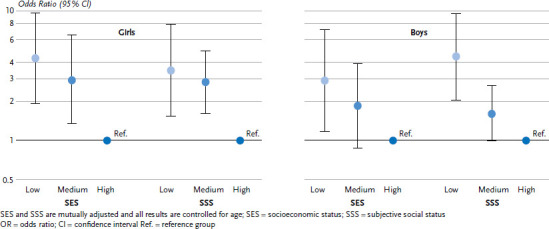
Associations of objective socioeconomic status (SES) and subjective social status (SSS) with general health (fair to very bad) in the 11-17 age group. Results of binary logistic regression models (OR with 95% CI, n=3,080 girls, n=2,808 boys) Source: KiGGS Wave 2 (2014-2017)

Figure 4 shows the odds ratios for fair to very bad general health by objective and subjective status. SES and SSS are simultaneously added to the regression models. As the results show, both a low SES and a low SSS remain independently associated with fair to very bad general health after mutual adjustment. The associations are slightly weaker than when considering them separately (Table 5 and Table 6). Although the two status indicators correlated with one another, both show independent associations with the general health of girls and boys in the 11 to 17 age group.

## 4. Discussion

Health monitoring at the Robert Koch Institute regularly provides data on the health of children, adolescents and adults and has in the past few years contributed significantly to improving the data basis for epidemiologic research and health reporting in Germany. This also applies to social epidemiological research and its focus on the relationship between socioeconomic status and health [47]. The conceptual development and use of an index that serves to measure socioeconomic status (SES index) across all health surveys, are therefore important elements considering the future challenges for the analysis of trends over time and longitudinal analyses on the effects of socioeconomic status on health and the course of diseases. To increase the index’s international comparability, the measurement of socioeconomic variables and their categorisation apply internationally established methods and instruments such as the CASMIN classification for school education and occupational qualifications [32] or the ISEI index for occupational status [33]. Using equivalised disposable income instead of household net income takes account of national and international recommendations to consider household size and member structure when assessing the income level of study participants [35].

Data on education, occupation and income was converted to metric scales. By dividing the SES index into quintiles, this allows for a distribution-based delimitation of socioeconomic groups. For health monitoring, the groups were split into high and low (20%, i.e. first and fifth quintile) and the medium status group (second to fourth quintile) comprising of 60% of the population. An analysis of all five socioeconomic groups (first to fifth quintile) is however also conceivable, and depends on the research question, as would be a division into tertiles or quartiles. The concept of relative social and health inequality provides the conceptual basis for a distribution-based distinction of socioeconomic groups. This rests on the assumption that belonging to the least or most socioeconomically disadvantaged group remains a relevant health determinant even when the overall wealth of a society increases and living conditions improve. For the analysis of trends over time, this means that at any specific point in time the 20% of the population facing the greatest socioeconomic disadvantages are compared with the 20% of the population with the greatest socioeconomic advantages, regardless of the overall levels of socioeconomic resources at that time.

A composite multidimensional SES index is suitable for describing the extent and development of health inequalities. An index based on a clear concept and simple operationalisation through three socioeconomic groups makes an analysis of the relationship between socioeconomic status and health understandable to a broad audience. Analyses based on the SES index therefore fulfil an important function in transferring the results to the realms of politics and practice. On the other hand, SES index-based analyses of health inequalities have only limited explanatory power regarding broader explanations or the definition of specific target groups for interventions. To this end, analyses based on the individual indicators education, occupation and income are more informative. They provide better conclusions for example on material living conditions, social participation or health-relevant attitudes and behaviours [17, 48].

An analysis of subjective social status, which was first surveyed in KiGGS Wave 2, can provide further insights. In line with international research, the results presented as examples for general health, reveal the clear association between SSS and child and adolescent health [21-23, 40, 49]. This relationship remains evident even when the SES index is also taken into account in multivariate analysis. This points to the need to assess SSS in addition to objective SES indicators in surveys on child and adolescent health. This would create a perspective on aspects of the socioeconomic conditions of families that a mere look at objective indicators such as education, occupation and income cannot provide. Income says nothing about whether a family is over-indebted, which is, nonetheless, a dimension of socioeconomic status. However, children and adolescents from these families probably experience the financial difficulties daily, and this then will reflect in their subjective perception of the family’s social status. Moreover, this can make the beliefs, values and attitudes of adolescents about social inequalities and the socioeconomic situation of their families come into effect. For example, children and adolescents may give greater weight to particular socioeconomic factors than others, if they consider them to be particularly important for the living conditions of their families. Surveys limited to traditional objective SES indicators are blind to such subjective factors. Nonetheless, these subjective factors may be related to feelings of shame, injustice, envy or a sense of inferiority, disadvantage and deprivation that can impact on health and well-being. Hence, the concept of SSS opens up a number of new perspectives for advances in research into health inequality and is a promising extension to objective SES indicators in epidemiological studies.

## Key statements

- Children and adolescents from socially disadvantaged families are more likely to have health problems and unfavourable health behaviours.
- The socioeconomic status (SES) index is generated as a household characteristic based on parental levels of education, occupational status and income.
- Subjective social status (SSS) describes the individual perception of objective living conditions.
- Children and adolescents from families with low SES or low SSS have poorer general health than their better-off peers.
- The SES index and SSS each are individually associated with the general health of children and adolescents.
- The established SES index has an important function in transferring the results on child and adolescent health inequality to the political sphere and practice.

## Figures and Tables

**Table 1:** Family socioeconomic status in KiGGS Wave 2 (n=7,426 girls, n=7,381 boys) Source: KiGGS Wave 2 (2014-2017)

Name of category	Quintile of SES	Lowest point score	Highest point score	Weighted percentage
Low	1^st^ quantile	3.2	8.7	20.1%
Medium	2^nd^ quantile	8.8	11.3	20.1%
	3^rd^ quantile	11.4	13.7	20.5%
	4^th^ quantile	13.8	16.9	19.4%
High	5^th^ quantile	17.0	21.0	20.0%

SES = socioeconomic status

**Table 2:** Correlation coefficients for the relationship between the SES index score and the education, occupation and income subscores (n=7,426 girls, n=7,381 Boys) Source: KiGGS Wave 2 (2014-2017)

Indicator		(1)	(2)	(3)	(4)	(5)	(6)
(1)	SES index score	1.00					
(2)	SES quintile	0.97	1.00				
(3)	SES groups	0.89	0.90	1.00			
(4)	SES subscore education	0.85	0.82	0.77	1.00		
(5)	SES subscore occupation	0.85	0.82	0.76	0.64	1.00	
(6)	SES subscore income	0.83	0.82	0.74	0.52	0.54	1.00

SES = socioeconomic status

**Table 3:** Mean and standard deviation of subjective social status ratings in the 11 to 17 age group (n=3,105 girls, n=2,822 boys) Source: KiGGS Wave 2 (2014-2017)

	Girls (M (SD))	Boys (M (SD))
**Total**	6.23 (1.37)	6.30 (1.39)
**Age**		
11-13	6.31 (1.39)	6.32 (1.38)
14-17	6.17 (1.36)	6.29 (1.39)
**Objective SES**		
Low	5.52 (1.52)	5.41 (1.47)
Medium	6.24 (1.24)	6.32 (1.26)
High	7.04 (1.16)	7.16 (1.12)

M = mean; SD = standard deviation; SES = socioeconomic status

**Table 4:** Correlation coefficients for the relationship between subjective social status and objective indicators of socioeconomic status in the 11 to 17 age group (n=3,105 girls, n=2,822 boys) Source: KiGGS Wave 2 (2014-2017)

Indicator	Girls (SSS)	Boys (SSS)
SES index score	0.39	0.42
SES subscore education	0.30	0.29
SES subscore occupation	0.28	0.32
SES subscore income	0.36	0.42

SES = socioeconomic status (objective); SSS = subjective social status

**Table 5:** Associations between socioeconomic status and general health (fair to very bad) in the 3 to 17 age group. Results of binary logistic regression models (OR with 95% CI and *p*-value, n=6,650 girls, n=6,610 boys) Source: KiGGS Wave 2 (2014-2017)

	Low SES	Medium SES	High SES
	OR (95%-CI) *p*-value	OR (95%-CI) *p*-value	
Girls*			
3 – 10	5.14 (2.19-12.09) 0.00	2.88 (1.35-6.13) 0.01	Ref.
11 – 17	7.15 (3.35-15.25) 0.00	4.27 (1.98-9.21) 0.00	Ref.
Total	6.28 (3.71-10.62) 0.00	3.61 (2.13-6.14) 0.00	Ref.
Boys*			
3 – 10	5.97 (3.10-11.50) 0.00	3.00 (1.72-5.25) 0.00	Ref.
11 – 17	5.17 (2.17-12.30) 0.00	2.34 (1.09-5.02) 0.03	Ref.
Total	5.57 (3.18-9.76) 0.00	2.65 (1.60-4.42) 0.00	Ref.
Total**			
3 – 10	5.68 (3.22-10.02) 0.00	2.96 (1.88-4.66) 0.00	Ref.
11 – 17	5.89 (3.25-10.69) 0.00	3.05 (1.77-5.24) 0.00	Ref.
Total	5.83 (3.87-8.78) 0.00	3.01 (2.10-4.32) 0.00	Ref.

SES = socioeconomic status; OR = odds ratio; CI = confidence interval; Ref. = reference group

* adjusted for age

** adjusted for age and gender

**Table 6:** Associations between subjective social status and general health (fair to very bad) in the 11 to 17 age group. Results of binary logistic regression models (OR with 95% CI and *p*-value, n=3,090 girls, n=2,817 boys) Source: KiGGS Wave 2 (2014-2017)

	Girls (11-17)*	Boys (11-17)*	Total (11-17)^**^
	OR (95%-CI) *p*-value	OR (95%-CI) *p*-value	OR (95%-CI) *p*-value
Low SSS (1-4)	4.99 (2.30-10.87) 0.00	6.02 (2.84-12.78) 0.00	5.57 (3.15-9.85) 0.00
Medium SSS (5-6)	3.55 (2.07-6.07) 0.00	1.86 (1.12-3.11) 0.02	2.57 (1.74-3.79) 0.00
High SSS (7-10)	Ref.	Ref.	Ref.

SSS = subjective social status; OR = odds ratio; CI = confidence interval; Ref. = reference group

* adjusted for age

** adjusted for age and gender

## Appendix

**Annex Table 1 (in german):** Questions on the operationalisation of socioeconomic status in KiGGS Wave 2 – parent questionnaire Source: KiGGS Wave 2 (2014-2017)

Bereich	Frage	Antwortkategorien
	*Bei den folgenden Fragen, die Vater und Mutter betreffen, meinen wir die Personen, die mit dem Kind in einem Haushalt leben. Mit der Bezeichnung „Mutter“ oder „Vater“ sind auch diejenigen Personen gemeint, die an die Stelle der leiblichen Eltern treten, z. B. Lebenspartnerin des Vaters, Stiefvater o. a.*	
**Bildung**	Welchen höchsten allgemeinbildenden Schulabschluss haben Sie?	Noch keinen Abschluss (noch Schüler)
		Abschluss nach höchstens 7 Jahren Schulbesuch
		Haupt-/Volksschule
		Realschule/Mittlere Reife/Mittlerer Schulabschluss (MSA)/Polytechnische Oberschule (POS)
		Abitur, allgemeine oder fachgebundene Hochschulreife, erweiterte Oberschule (EOS), Fachhochschulreife/Fachoberschule
		Anderer Schulabschluss (z. B. im Ausland erworben)
	Welchen höchsten beruflichen Abschluss haben Sie?	Keinen Abschluss, noch in beruflicher Ausbildung, z. B. Student/in, AZUBI, Berufsvorbereitungsjahr, Praktikant/in
		Keinen Berufsabschluss und nicht in Ausbildung
		Lehre, also beruflich-betriebliche Ausbildung
		Ausbildung an Berufsfachschule, Handelsschule, also beruflich-schulische Ausbildung
		Fachschule, z. B. Meister-, Technikerschule, Berufs- oder Fachakademie
		Fachhochschule, Ingenieurschule
		Universität oder Hochschule
		Anderen Ausbildungsabschluss (z. B. im Ausland erworben)
**Beruf**	Sind Sie derzeit…	…Vollzeit erwerbstätig
		…Teilzeit erwerbstätig
		…Geringfügig erwerbstätig
	Welche berufliche Stellung haben Sie in Ihrer Haupterwerbstätigkeit? *Wenn Sie derzeit nicht oder nicht mehr berufstätig sind, nennen Sie bitte die berufliche Stellung, die Sie zuletzt innehatten.*	Angestellte/r
		Arbeiter/in
		Beamtin/Beamter (auch Anwärter/in)
		Landwirt/in im Haupterwerb
		Selbstständig erwerbstätig mit Mitarbeitern
		Selbstständig erwerbstätig ohne Mitarbeiter
		Mithelfende/r Familienangehörige/r (unbezahlt)
		Auszubildende/r (auch Praktikant/in, Volontär/in)
		Freiwillig Wehrdienst- oder Bundesfreiwilligendienstleistende/r
		Freiwilliges soziales/ökologisches/kulturelles Jahr
		Noch nie erwerbstätig gewesen
**Beruf**	Nehmen Sie eine Führungsaufgabe wahr, d. h. sind Sie Mitarbeitern/Mitarbeiterinnen gegenüber weisungsbefugt, die keine Auszubildenden sind?	Ja, als Führungskraft (mit Entscheidungsbefugnis über Personal, Budget und Strategie)
		Ja, als Aufsichtskraft (Anleiten und Beaufsichtigen von Personal, Verteilen und Kontrollieren von Arbeit)
		Nein
**Einkommen**	Wie hoch ist in etwa das monatliche NettoEinkommen Ihres Haushalts insgesamt? *Bitte zählen Sie die monatlichen Einkommen aller Haushaltsmitglieder (einschließlich Elterngeld, Kindergeld usw.) nach Abzug von Steuern und Sozialabgaben zusammen.*	*Betrag als offene Angabe in EURO*
		(*Bei Verweigerung*) *Einkommen in Kategorien erfassen*
	Wie viele Personen leben ständig in Ihrem Haushalt, Sie selbst mit eingerechnet?	*Anzahl der Personen*
	Wie viele Personen in Ihrem Haushalt sind jünger als 14 Jahre?	*Anzahl der Personen unter 14 Jahren*

**Annex Table 2 (in German):** Basis to calculate the SES index in KiGGS Wave 2 Source: KiGGS Wave 2 (2014-2017)

Punkte		Bildung	Beruf	Einkommen
von	bis unter	Schulische und berufliche Qualifikation nach CASMIN-Klassifikation	Berufliche Stellung nach EHIS (Berufl. Stellung, Führungsaufgaben)	Nettoäquivalenzeinkommen
1,0	1,5	1a (Kein schulischer Abschluss und kein beruflicher Abschluss) 1,0 Pkt.	Landwirt im Haupterwerb: 1,0 Pkt.	78 EUR – 609 EUR: 1,0 Pkt.
1,5	2,0	1b (Abschluss nach höchstens 7 Jahren Schulbesuch/Haupt-/Volksschule und kein beruflicher Abschluss) 1,7 Pkt.	–	610 EUR – 821 EUR: 1,5 Pkt.
2,0	2,5	–	Arbeiter o. Führungs-/Aufsichtstätigkeit: 1,9 Pkt.	822 EUR – 960 EUR: 2,0 Pkt.
			Arbeiter o. n. A.: 2,0 Pkt.	
2,5	3,0	2b (Realschule/Mittlere Reife/Mittlerer Schulabschluss/Polytechnische Oberschule und kein beruflicher Abschluss) 2,8 Pkt.	Arbeiter Aufsichtskraft/Führungskraft: 2,7 Pkt.	961 EUR – 1.091 EUR: 2,5 Pkt.
3,0	3,5	1c (Kein Abschluss von Realschule/Mittlere Reife/Mittlerer Schulabschluss/Polytechnische Oberschule und abgeschlossene Lehre, also berufliche-betriebliche Ausbildung) 3,0 Pkt.	–	1.092 EUR – 1.221 EUR: 3,0 Pkt.
3,5	4,0	2a (Realschule/Mittlere Reife/Mittlerer Schulabschluss/Polytechnische Oberschule und abgeschlossene Lehre, also berufl.-betriebl. Ausbildung) 3,6 Pkt.	Sonstige: 3,8 Pkt.	1.222 EUR – 1.344 EUR: 3,5 Pkt.
		2c-gen (Abitur, allgemeine/fachgebundene Hochschulreife, Erweiterte Oberschule, Fachhochschulreife/Fachoberschule und kein beruflicher Abschluss) 3,7 Pkt.		
4,0	4,5	–	–	1.345 EUR – 1.454 EUR: 4,0 Pkt.
4,5	5,0	2c-voc (Abitur, allg./fachgebundene Hochschulreife, Erweiterte Oberschule, Fachhochschulreife/Fachoberschule und beruflicher Abschluss) 4,8 Pkt.	Angestellter o. Führungs-/Aufsichtstätigkeit: 4,4 Pkt.	1.455 EUR – 1.600 EUR: 4,5 Pkt.
			Angestellter o. n. A.: 4,7 Pkt.	
			Angestellter Aufsichtskraft: 4,8 Pkt.	
5,0	5,5	–	Selbstständig ohne Mitarbeiter: 5,1 Pkt.	1.601 EUR – 1.762 EUR: 5,0 Pkt.
5,5	6,0	–	Selbstständig mit Mitarbeitern: 5,5 Pkt.	1.763 EUR – 1.971 EUR: 5,5 Pkt.
6,0	6,5	3a (Abschluss Fachhochschule, Ingenieurschule) 6,1 Pkt.	Angestellter Führungskraft: 6,1 Pkt.	1.972 EUR – 2.260 EUR: 6,0 Pkt.
6,5	7,0	–	–	2.261 EUR – 2.833 EUR: 6,5 Pkt.
7,0		3b (Abschluss Universität oder Hochschule) 7,0 Pkt.	Beamte (alle Gruppen) 7,0 Pkt.	2.834 EUR u. m.: 7,0 Pkt.

CASMIN = Comparative Analyses of Social Mobility in Industrial Nations; o. = ohne; o. n. A. = ohne nähere Angabe; u. m. = und mehr; Pkt. = Punkte; EUR = Euro

**Annex Table 3 (in German):** Questions to operationalise subjective social status in KiGGS Wave 2 – questionnaire for children and adolescents aged 11 to 17 Source: KiGGS Wave 2 (2014-2017)

Bereich	Frage	Antwortkategorien
**Subjektiver Sozialstatus**	**Wie siehst du die Situation deiner Familie?**	[Bild einer Leiter mit 10 Sprossen, die den Werten 1 – 10 zugeordnet werden]
	*Stelle dir bitte vor, dass diese Leiter den Aufbau der Gesellschaft in Deutschland darstellt.*	
	*Ganz oben stehen die Menschen mit dem meisten Geld, der höchsten Bildung und den besten Jobs. Ganz unten stehen die Menschen mit dem wenigsten Geld, der niedrigsten Bildung und den schlechtesten Jobs oder ohne Job.*	
	*Nun denke an deine Familie.* **Was denkst du, auf welcher Sprosse würde deine Familie stehen? Bitte kreuze einen Kreis neben der Leiter an.**	
